# Diffusion probabilistic *versus* generative adversarial models to reduce contrast agent dose in breast MRI

**DOI:** 10.1186/s41747-024-00451-3

**Published:** 2024-05-01

**Authors:** Gustav Müller-Franzes, Luisa Huck, Maike Bode, Sven Nebelung, Christiane Kuhl, Daniel Truhn, Teresa Lemainque

**Affiliations:** Department of Diagnostic and Interventional Radiology, Medical Faculty, RWTH Aachen University, Aachen, Germany

**Keywords:** Artificial intelligence, Breast neoplasms, Contrast media, Machine learning, Magnetic resonance imaging

## Abstract

**Background:**

To compare denoising diffusion probabilistic models (DDPM) and generative adversarial networks (GAN) for recovering contrast-enhanced breast magnetic resonance imaging (MRI) subtraction images from virtual low-dose subtraction images.

**Methods:**

Retrospective, ethically approved study. DDPM- and GAN-reconstructed single-slice subtraction images of 50 breasts with enhancing lesions were compared to original ones at three dose levels (25%, 10%, 5%) using quantitative measures and radiologic evaluations. Two radiologists stated their preference based on the reconstruction quality and scored the lesion conspicuity as compared to the original, blinded to the model. Fifty lesion-free maximum intensity projections were evaluated for the presence of false-positives. Results were compared between models and dose levels, using generalized linear mixed models.

**Results:**

At 5% dose, both radiologists preferred the GAN-generated images, whereas at 25% dose, both radiologists preferred the DDPM-generated images. Median lesion conspicuity scores did not differ between GAN and DDPM at 25% dose (5 *versus* 5, *p* = 1.000) and 10% dose (4 *versus* 4, *p* = 1.000). At 5% dose, both readers assigned higher conspicuity to the GAN than to the DDPM (3 *versus* 2, *p* = 0.007). In the lesion-free examinations, DDPM and GAN showed no differences in the false-positive rate at 5% (15% *versus* 22%), 10% (10% *versus* 6%), and 25% (6% *versus* 4%) (*p* = 1.000).

**Conclusions:**

Both GAN and DDPM yielded promising results in low-dose image reconstruction. However, neither of them showed superior results over the other model for all dose levels and evaluation metrics. Further development is needed to counteract false-positives.

**Relevance statement:**

For MRI-based breast cancer screening, reducing the contrast agent dose is desirable. Diffusion probabilistic models and generative adversarial networks were capable of retrospectively enhancing the signal of low-dose images. Hence, they may supplement imaging with reduced doses in the future.

**Key points:**

• Deep learning may help recover signal in low-dose contrast-enhanced breast MRI.

• Two models (DDPM and GAN) were trained at different dose levels.

• Radiologists preferred DDPM at 25%, and GAN images at 5% dose.

• Lesion conspicuity between DDPM and GAN was similar, except at 5% dose.

• GAN and DDPM yield promising results in low-dose image reconstruction.

**Graphical Abstract:**

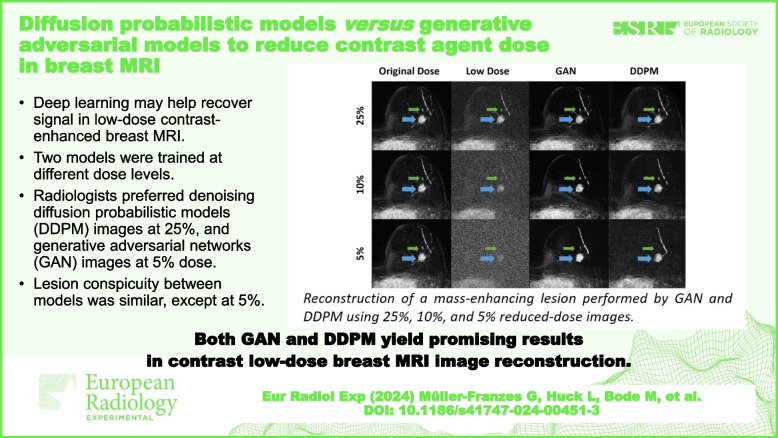

**Supplementary Information:**

The online version contains supplementary material available at 10.1186/s41747-024-00451-3.

## Background

Current mammography-based breast cancer screening strategies miss lesions in women with extremely dense breasts. Thus, the current recommendation of the European Society of Breast Imaging (EUSOBI) calls for the introduction of magnetic resonance imaging (MRI)-based screening for these women [1]. Breast MRI relies on the dynamic contrast-enhanced (DCE) sequence, which acquires one dynamic phase before and several dynamic phases after bolus injection of gadolinium-based contrast agent (GBCA). GBCA accumulation visualizes perfusion associated with highly neoangiogenic lesions and leaky vasculature [2]. Malignant breast lesions, characterized by fast enhancement, can be detected on the first postcontrast dynamic scan [3]. Especially in a screening context, a lower GBCA dose is desirable, not only for patients with an impaired renal function, but also to minimize Gd deposition in the brain, shown to occur with linear GBCA [4], but less likely with macrocyclic GBCA [5]. Yet, dose reduction decreases contrast-to-noise ratio (CNR) in the subtraction images, as less GBCA stands against an unchanged noise level. Although previous reports suggest that reduced GBCA doses effectively depict breast lesions [6, 7], a high CNR remains advantageous.

Deep learning (DL) may help reduce GBCA dose without sacrificing image quality [8–13]. In the context of breast imaging, we have previously employed generative adversarial networks (GAN) to retrieve high-dose from low-dose virtual subtraction images [8]. To obtain data for training and testing purposes, the CNR of the original images was artificially reduced by increasing the noise level. Likewise, recovering the dose required denoising the low-dose images. Previously, the GAN was tailored to a fixed GBCA dose of 25% [8]. In clinical application, the GBCA dose employed by different hospitals and in the research setting might vary. It is unclear, if a GAN trained on a specific CNR level (*i.e.*, GBCA dose) can be equally applied to any CNR and while it is technically possible to train separate GAN models for different input CNR levels, a more flexible solution is useful.

This is where denoising diffusion probabilistic models (DDPM) come into play. During training, successive amounts of noise are added to an input image along a Markov chain of “time steps” *t* and the DDPM is trained to revert this process step-by-step. In the extreme setting of pure noise (*i.e.*, *t* is infinite) DDPMs can even generate realistic-looking images from no information [14]. However, in our study, we employ the ingrained denoising capability of DDPMs on existing, yet noisy images. In detail, this study employed a DDPM to retrieve original-dose from virtual low-dose breast DCE-MRI subtraction images at different input noise levels and compared it against GAN models trained for the same purpose. We hypothesize that the DDPM is more flexible in the sense that it can denoise low-dose input images of different dose levels, and that it outperforms GANs across a range of input GBCA doses between 5 and 25% of the original dose.

## Methods

### Study design and data set

**Fig. 1:**
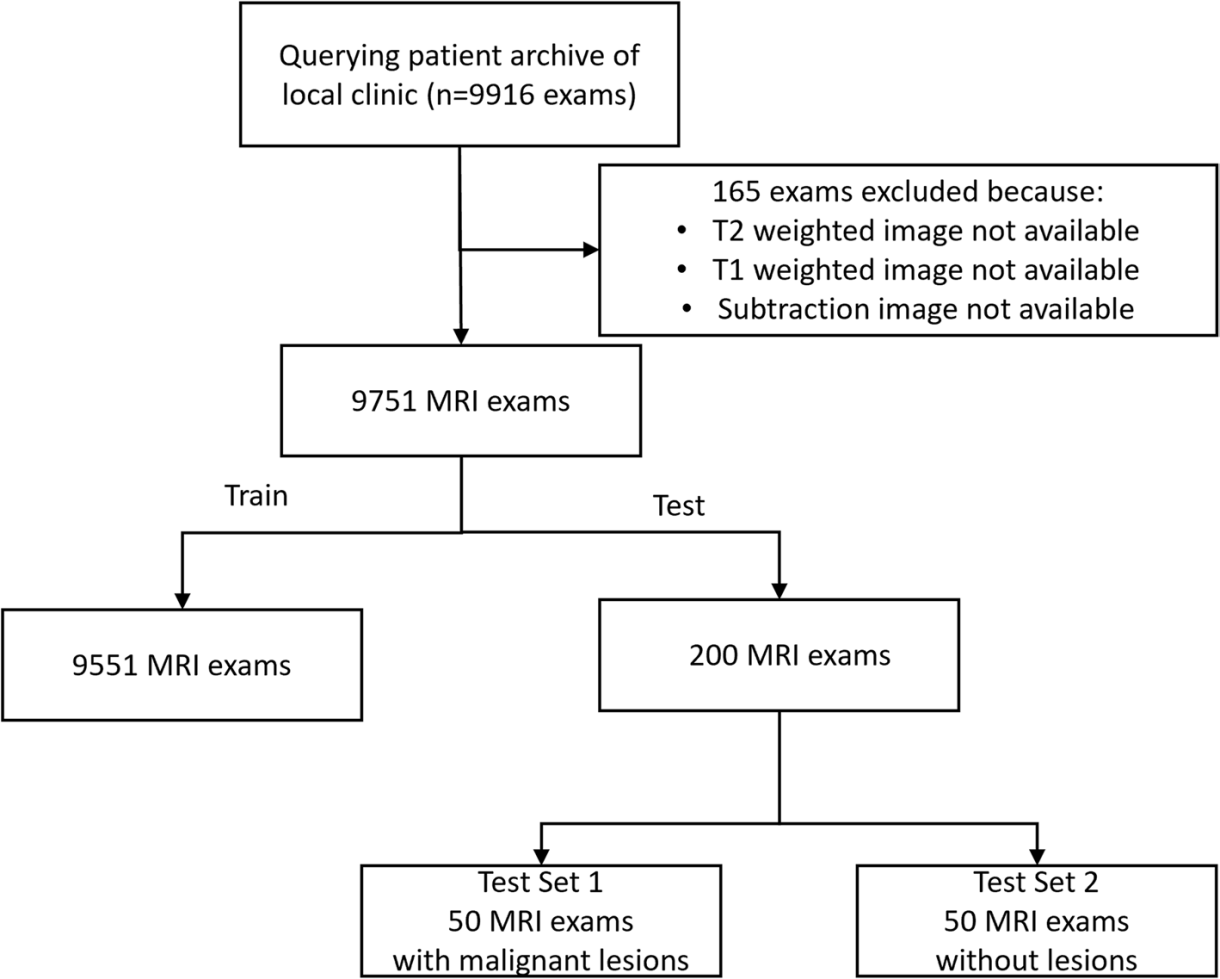
Flowchart of the study, from initial retrieval to final study cohort. Please note that the initial selection of 200 test examinations was chosen to match the training set with previous work. *MRI* Magnetic resonance imaging

For this retrospective study, institutional review board approval was obtained (EK028/19) prior to study initiation. Figure 1 describes the retrieval of data acquired between January 2010 and November 2019 from the local database, resulting in a final dataset of 9,751 breast MRI examinations. Following the splitting as defined in [8], 9,551 examinations, *i.e.*, 19,102 single-sided breast examinations, were assigned to the training dataset. Please note that we have exclusively used the single-sided breast examinations and no additional patient-level information to increase the number of training images available to us and to focus on the question whether all relevant information is still encoded in the image after reduced contrast agent administration. Two hundred patient-stratified examinations were held out separately for model testing. Out of the latter, two test sets were composed for this study. Test set 1 included 50 single-sided breast examinations with malignant lesions. Test set 2 included 50 single-sided breast examinations without lesions.

### Image acquisition

MRI was performed according to a standardized protocol [15] at 1.5 T (Philips Achieva or Philips Ambition, Best, The Netherlands) in prone position, using a four-channel breast coil (Invivo, Gainesville, FL) with compression paddles (Noras, Höchberg, Germany) immobilizing the breasts along the cranio-caudal direction. Axial bilateral T2-weighted fast spin echo images were acquired followed by an axial bilateral T1-weighted gradient echo DCE series, for which one and four dynamic scans were performed prior to and after the injection of GBCA (Gadovist, Bayer, Leverkusen, Germany). First postcontrast subtraction images were generated on the scanner workstation by subtracting the baseline image from the first dynamic scan after contrast injection. Please note that field strength, breast coil, breast compression technique and the protocol remained consistent over the period of data acquisition. Scan parameters can be found in Supplementary Table S1.

### Image preprocessing

To standardize the input for the DL models, we first resampled all subtraction images to an in-plane resolution of 0.64 × 0.64 mm^2^ and cropped them within a rectangular bounding box including the musculus pectoralis and both nipples. Subsequently, the cropped volume was divided into the left and right breast along the median plane. The single-sided breast images were cropped and zero-padded to a uniform size of 256 × 224 voxels. They will be referred to as original subtraction images in the following.

### Generation of noisy test images

Mathematically, the CNR of a subtraction image can be decreased in two ways, that is, by decreasing the GBCA dose or by increasing the amount of noise. Hence, virtual low-dose subtraction images were simulated by adding white Gaussian noise to the original subtraction images. Details of the process have been described previously [8]. To train and test the performance of DL models at different low doses, virtual subtraction images corresponding to 25%, 10%, and 5% GBCA doses were calculated.

### DL models for GBCA dose reduction

Two types of DL models (DDPM and GAN) were implemented and trained for synthetic image generation.

**Fig. 2:**
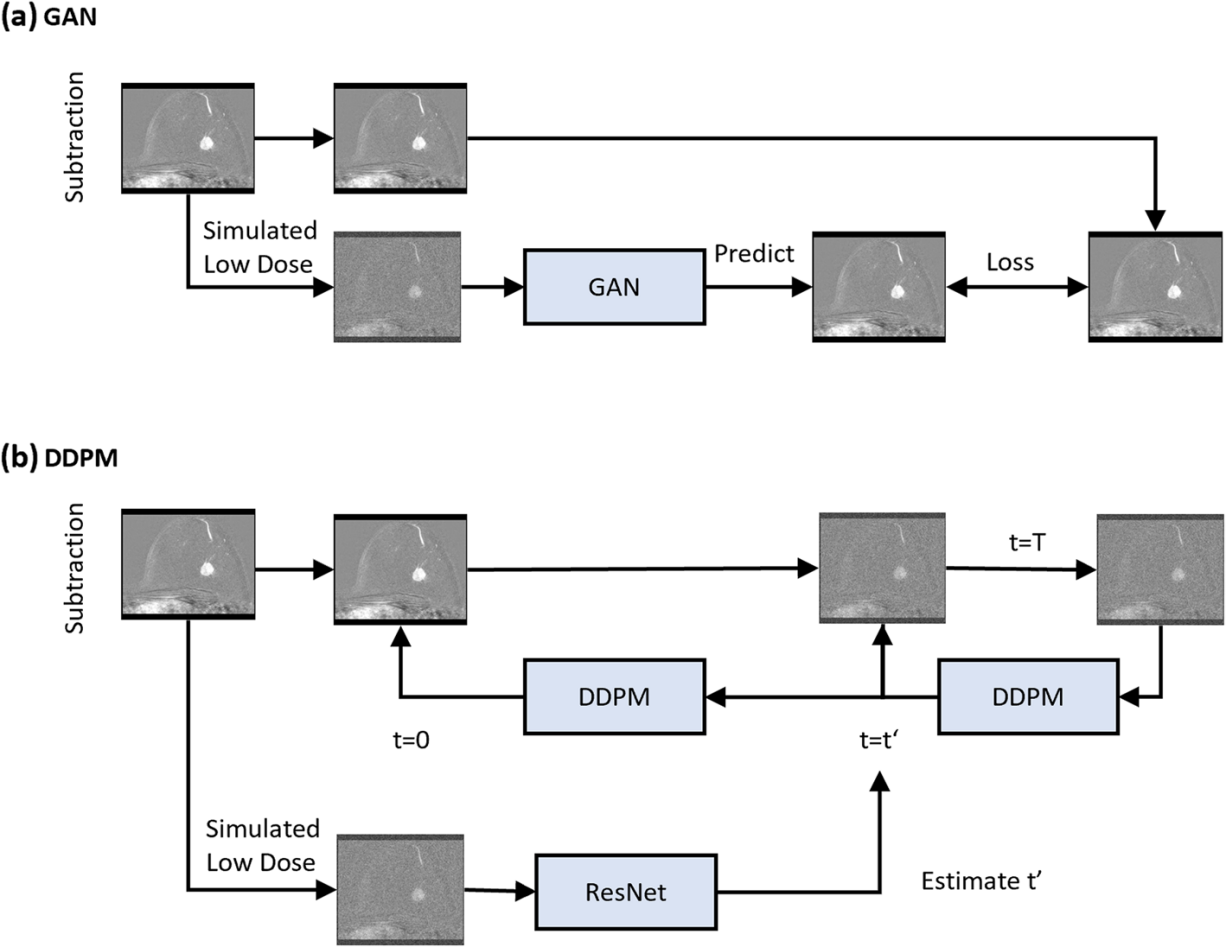
Illustration of the architecture of GAN and DDPM deep learning networks.** a** The GAN-25, GAN-10, or GAN-5 model receives the simulated low-dose (25%, 10%, or 5%) image, respectively, and predicts the full-dose image. During training, the predicted and real full-dose images are compared via a loss function. **b** The DDPM model is trained to iteratively denoise images at different stages of noise. A ResNet was employed to estimate the required number of time steps t′ that are required for the trained DDPM to denoise a given low-dose subtraction image. *DDPM* Denoising diffusion probabilistic model, *GAN* Generative adversarial network, *ResNet* Residual network

For the training of the DDPM [14], a cosine-noise schedule [16] with *t* = 1,000 steps was employed to add Gaussian noise to the original subtraction images. The L1 loss and the AdamW optimizer [17] with a learning rate of 0.0001 were used to train the DDPM. For inference, *i.e*., obtaining a denoised high-dose image from a virtual low-dose one, the noise level in the latter needed to be estimated to determine the corresponding starting point (*t*) in the Markov chain between the entirely noisy (t = T) and the denoised image (*t* = 0). For this purpose, we trained a ResNet-34 [18] separately from the DDPM. Controlled amounts of noise were added to original images using the DDPM’s cosine-noise scheduler, with the training objective to estimate the time *t*′ that corresponded to the noise level in the virtual low-dose images (Fig. 2). The L1 loss between estimated *t*′ and true *t* was minimized by the AdamW optimizer with a learning rate of 0.0001.

We used the Pix2PixHD GAN model [19] to reconstruct high-dose subtraction images from the low-dose images. Three distinct models (referred to as GAN-25, GAN-10, and GAN-5) were trained, using the low-dose images at reduced doses of 25%, 10%, or 5% next to the original ones as input, respectively (Fig. 2). The Adam optimizer [20] with a learning rate of 0.002 [21] was employed.

### Synthetic image generation

The virtual subtraction images corresponding to 25%, 10%, and 5% GBCA dose were first fed into the ResNet, which estimated the required number of time steps *t*′ to restitute the original-dose image, and then, together with the such identified *t*′, into the DDPM. The same virtual subtraction images were fed into the GAN-25, GAN-10, or GAN-5 models, so that the dose level of an input image and the trained model were in correspondence. In total, 150 DDPM-reconstructed and 150 GAN-reconstructed stacks of images were obtained per test set (*i.e.*, 50 for each model type and dose level, respectively), as well as maximum intensity projections (MIPs) along the axial direction.

### Image analysis

#### Qualitative image assessment by radiologists

Radiologic evaluations were performed by two radiologists with 6 and 7 years of experience in breast imaging via an in-house developed browser tool. In a first experiment, radiologists were presented with sets of three single-slice images that centrally bisected the enhancing lesion. Image 1 was the original subtraction image, while image 2 and image 3 were the GAN-reconstructed or DDPM-reconstructed ones. Readers were blinded to the nature of the model and to the input dose. Care was taken to present the three images with comparable windowing. The readers stated whether they preferred image 2 or image 3 as compared to the original and rated the conspicuity of the enhancing lesion on both reconstructed images on a Likert scale from 1 = poor to 5 = excellent, again with the original image as reference standard. In a second experiment, readers were presented with sets of two MIPs. MIP 1 was generated based on the original subtraction images, while MIP 2 was generated based on the DDPM- or the GAN-reconstructed images. Readers were asked to state if the synthetic MIP contained at least one lesion that was not visible in the original MIP.

#### Numerical image assessment

To quantitatively assess the similarity or dissimilarity between the real and synthetic images generated by the DDPM and GANs, we calculated four metrics: the structural similarity index measure (SSIM) [22], peak signal-to-noise ratio (PSNR), mean squared error (MSE) and learned perceptual patch similarity (LPIPS) [23]. Higher SSIM, higher PSNR, lower MSE, and lower LPIPS indicate higher agreement between two images.

### Statistical analysis

Statistical evaluations were performed by T.L. using R (v4.3.1, R Core Team, 2021). Per dose level, two-sided binomial tests were employed to evaluate if a reader’s preference for a model was statistically different from random choice. Median lesion conspicuity values were calculated per model (“GAN,” “DDPM”), dose level (5%, 10%, 25%), and reader. Lesion conspicuity was then assessed by means of ordinal mixed effects logistic regression, using a cumulative link mixed model (“clmm” from the “ordinal” package) with model, dose, and reader as fixed effects, while considering two-way interactions between model and dose, and the subject as random effect:

$$model<-clmm(score\sim dose\ast model+reader+(\mathit1\vert subject))$$

Score and dose were defined as ordinal variables with 5 and 3 levels, respectively, while reader, subject, and model were defined as nominal variables. Similarly, the effects of dose and model on the occurrence of false positive findings were assessed by mixed effects logistic regression:

$$model<-clmm(answer\sim dose\ast model+reader+(\mathit1\vert uid))$$

Here, answer constitutes a nominal variable with two levels. Mean SSIM, PSNR, MSE, LPIPS, and their SD were calculated per dose level and reader. For each metric, a linear mixed effect model (“lmer” from the “lme4” package) was fitted as follows:

$$model<-lmer(metric\sim dose\ast model+(\mathit1\vert subject)$$

Per fitted model, estimated marginal means were calculated using the “emmeans” package, on the basis of which pairwise post hoc tests were performed with Bonferroni correction to adjust for the multiple comparison problem. Please note that Bonferroni correction in R ceils at *p*-values of 1.000 [24]. Where applicable, pairwise comparison results were obtained as averaged over both readers. Throughout all statistical evaluations, a significance level of 0.05 was set. The sample size of the test sets was chosen in line with previous studies [8, 13, 25].

### Code availability

The code for the GAN model is publicly available at https://github.com/mueller-franzes/BreastMRI-Pix2PixHD and for the DDPM model at https://github.com/mueller-franzes/medfusion.

## Results

### Patient characteristics

Table 1 presents thr demographic details of the study cohort. Test set 1 included 50 examinations with 29 mass-enhancing and 21 non-mass-enhancing malignant lesions. The average lesion size was 24 ± 10 mm (mean ± standard deviation).

**Table 1:** Summary of demographic data for the training dataset and both test datasets

	Training set	Test set 1	Test set 2
Country	Germany	Germany	Germany
Patients	4,886	50	50
Examinations, total	9,551	50	50
Examinations, single-side	19,102	50	50
Sex	All women	All women	All women
Age (mean ± standard deviation)	56 ± 10	57 ± 10	56 ± 11

### Image quality decreases towards lower dose for both models

**Fig. 3:**
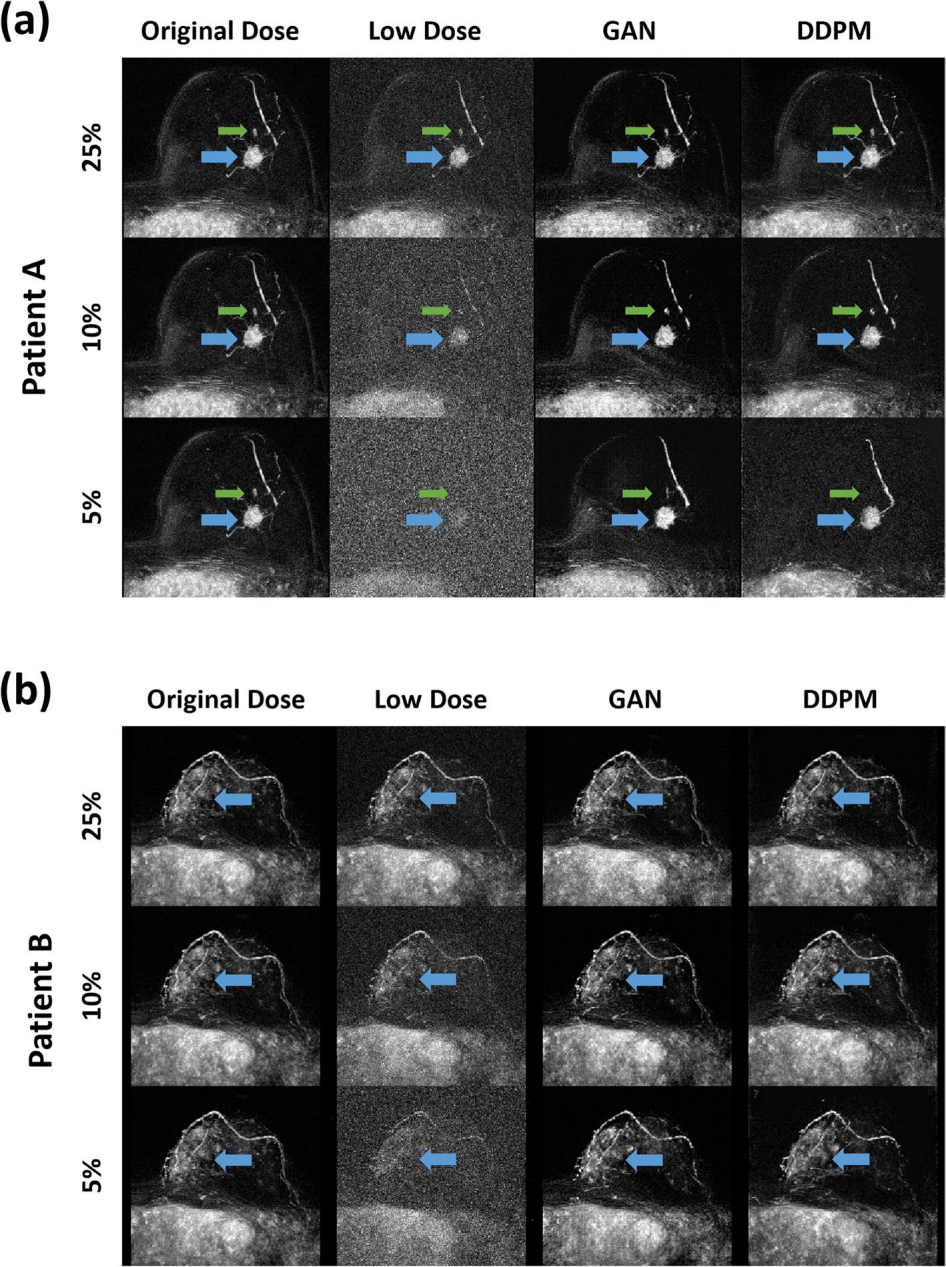
The reconstruction of a mass enhancing lesion (**a**) and a non-mass enhancing lesion (**b**) was performed by GAN and DDPM using 25%, 10%, and 5% reduced-dose images. Patient A was a 62-year-old woman with a triple-negative invasive breast cancer (blue arrow) in the left breast with a small satellite lesion ventral the main tumor (green arrow): no special type, Grade 3, stage pT1c (tumor size > 10 and ≤ 20 mm in diameter). Although the main lesion could be reconstructed even at 5% dose, details of its margin and the surrounding spicula are no longer discernible. The satellite lesion located ventral to the main tumor is still residual in the GAN 5% reconstruction, but no longer discernible in the DDPM 5% reconstruction. Vessels are no longer discernible in detail in any of the 10% and 5% reconstructions. Patient B was a 39-year-old woman with extensive segmental intermediate-grade ductal carcinoma *in situ* − DCIS (blue arrow) in the left breast. When comparing GAN 5% and DDPM 5%, the segmental enhancement appears marginally fainter in the DDPM 5% latter reconstruction. *DDPM* Denoising diffusion probabilistic model, *GAN* Generative adversarial network

Figure 3a shows GAN- and DDPM-reconstructed high-dose subtraction images of a 62-year-old woman with mass enhancement next to the original subtraction. Histology revealed a triple-negative invasive breast cancer in the left breast. Reducing the dose from 25 to 5% led to a decrease in the visual quality of reconstructed images for both models. Figure 3b shows an extensive segmental non-mass-enhancement in the left breast of a 39-year-old woman. Histology revealed an intermediate-grade ductal carcinoma *in situ*. The dose reduction from 25 to 5% led at most to a slight decrease in the visual image quality of the reconstructed images for both models.

Quantitative metrics confirmed the visual perception of decreased image quality with decreasing dose (Table 2). For example, SSIM of the DDPM decreased from 0.6 ± 0.1 to 0.43 ± 0.09 (mean ± standard deviation) (*p* < 0.001) as the dose decreased from 25 to 5%. Likewise, SSIM of the GAN decreased from 0.6 ± 0.1 to 0.4 ± 0.1 (*p* < 0.001). Accordingly, linear mixed models confirmed a significant influence of the dose on all four metrics. The type of model, *i.e.*, GAN or DDPM, reached a close-to-significant influence on modeling the SSIM (*p* = 0.059), and a significant one for modeling MSE, PSNR, and LPIPS. For complete statistical results, please refer to Supplementary Table S2.

**Table 2:** Quantitative metrics of DDPM and GAN reconstructed images

Metric	Dose	GAN	DDPM	*p*-value
SSIM	25	0.6 ± 0.1	0.6 ± 0.1	1.000
	10	0.5 ± 0.1	0.5 ± 0.1	1.000
	5	0.41 ± 0.09	0.43 ± 0.09	**0.002**
PSNR	25	26 ± 2	24 ± 4	**0.020**
	10	24 ± 4	21 ± 5	** < 0.001**
	5	22 ± 3	20 ± 5	**0.004**
MSE	25	70 ± 20	90 ± 20	** < 0.001**
	10	80 ± 20	100 ± 20	** < 0.001**
	5	90 ± 20	100 ± 20	0.090
LPIPS	25	0.2 ± 0.2	0.3 ± 0.2	0.240
	10	0.3 ± 0.1	0.4 ± 0.1	** < 0.001**
	5	0.3 ± 0.2	0.4 ± 0.1	** < 0.001**

Different metrics were calculated between the original image and reconstructed image. Scores are presented as mean value and standard deviation. *p*-values indicate selected pair-wise comparison results as obtained from mixed-effects linear regression and subsequent estimation of estimated marginal means. During pair-wise comparison, Bonferroni correction was applied. Significant *p*-values are marked in bold type. *DDPM* Denoising diffusion probabilistic model, *GAN* Generative adversarial network, LPIPS Learned perceptual image patch similarity, *MSE* Mean squared error, *PSNR* Peak-signal-to-noise ratio, *SSIM* Structural similarity index measure

### Readers prefer the DDPM at 25% and the GAN at 5% dose

**Fig. 4:**
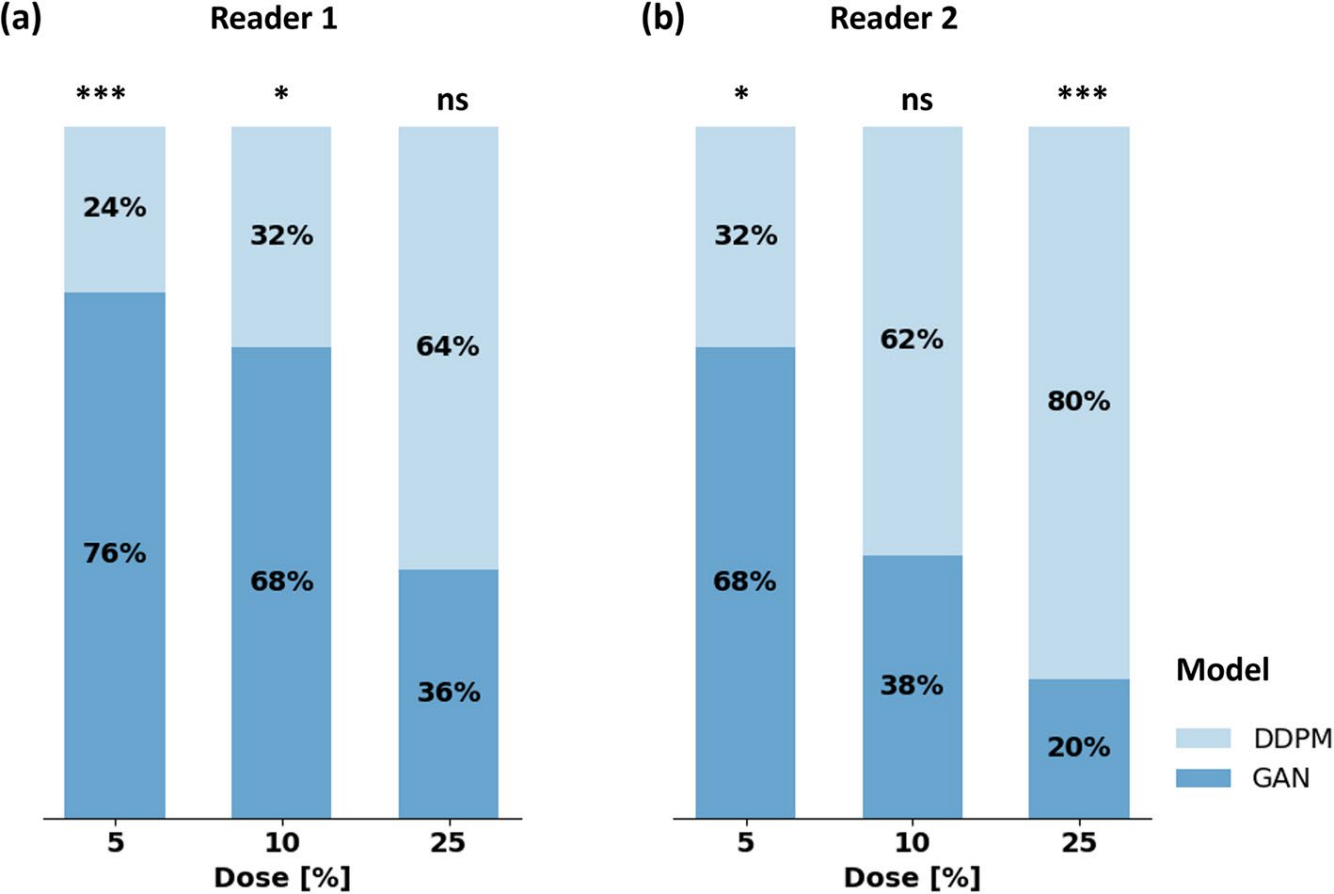
Model preference depending on the input dose. **a** Results for Reader 1; **b** results for Reader 2. The two-tailed binomial test was used to decide whether preference was different from random choice. Levels of statistical significance were stratified as “ns,” “*,” “**,” and “***” to indicate *p* > 0.05, 0.01 < *p* ≤ 0.05, 0.001 < *p* ≤ 0.01, and *p* ≤ 0.001. *DDPM* Denoising diffusion probabilistic model, *GAN* Generative adversarial network

At 25% dose, both readers preferred the DDPM-generated images (*p* = 0.060 and *p* < 0.001 for Reader-1 and Reader-2, respectively, with Reader-1’s preference being close-to, but not yet statistically significant), whereas at 5% dose, both readers preferred the GAN-generated images (*p* < 0.001 and *p* = 0.01). At 10% dose, reader preferences diverged (Fig. 4).

PSNR, MSE, and LPIPS indicated a closer agreement of the GAN-generated images with the original subtraction images compared to the DDPM-generated images, independent of the dose (Table 2), *i.e.*, higher PSNR and lower MSE and LPIPS. Pair-wise comparisons of these metrics between GAN and DDPM at the same dose levels were mostly significant. SSIM of GAN and DDPM were similar at 25% and 10%, except at 5%, where DDPM images yielded higher SSIM (*p* = 0.002).

### Comparable lesion conspicuity between both models, except at 5% dose

**Fig. 5:**
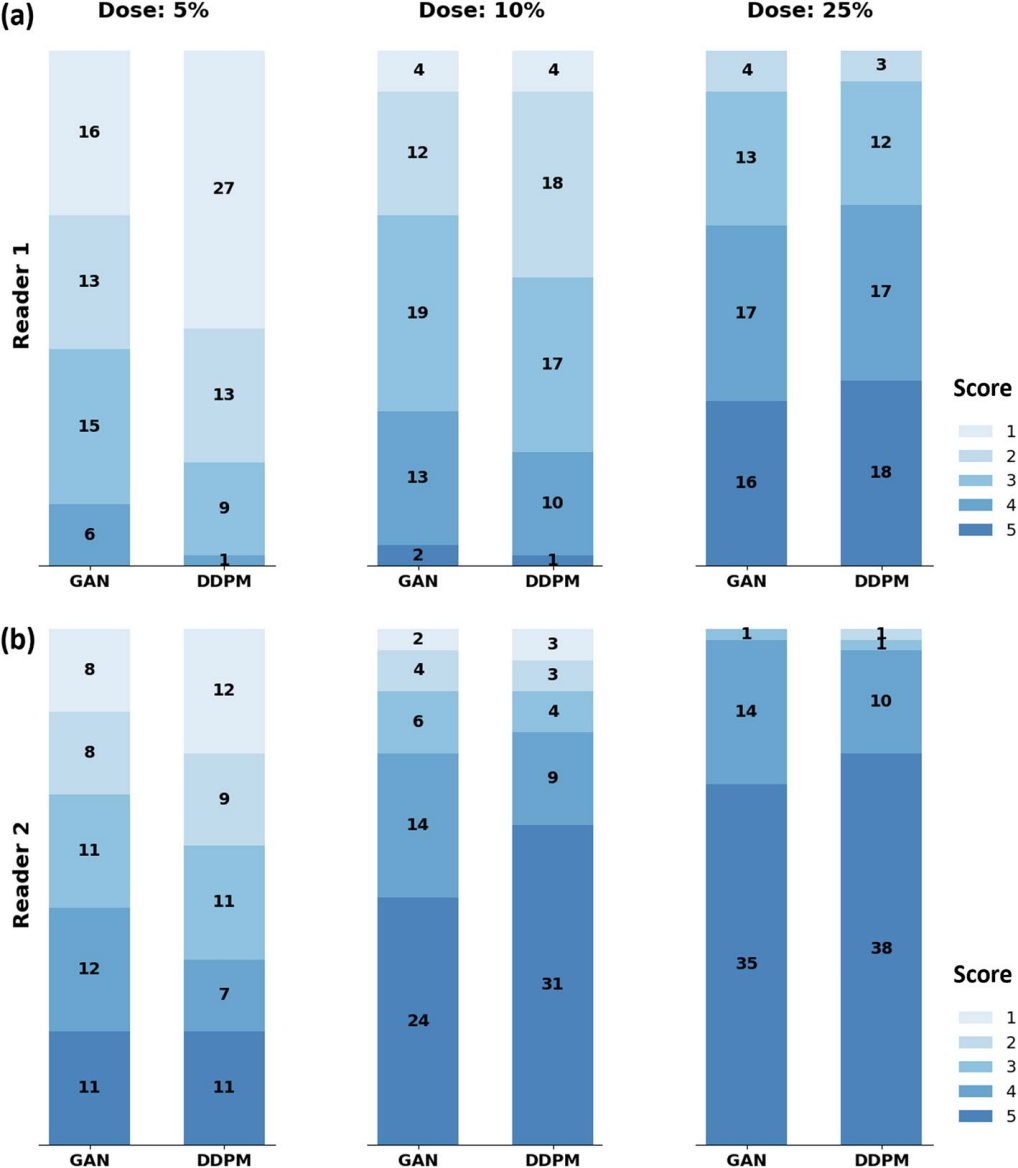
Lesion conspicuity scores for DDPM and GAN models. **a** Distribution of lesion conspicuity scores for Reader 1 and both models at 5%, 10%, and 25% of the original dose, respectively. **b** Corresponding results for reader 2. *DDPM* Denoising diffusion probabilistic model, *GAN* Generative adversarial network

Higher lesion conspicuity scores were assigned at higher input doses by both readers (Fig. 5). At 5% dose, both readers rated the lesion conspicuity higher for the GAN than for the DDPM (median score of 2 *versus* 1 for Reader-1, 3 *versus* 2 for Reader-2, Table 3). It should be noted that Reader-2 assigned overall higher lesion conspicuity scores than Reader-1. Ordinal logistic regression revealed dose and reader to have a significant effect on the score (both *p* < 0.001), but not the model type (*p* = 0.130). Supplementary Table S3 indicates complete statistical results. According to pair-wise post hoc tests (Table 2), conspicuity scores did not differ significantly between GAN and DDPM at 25% and 10%, but at 5% dose (*p* = 0.007).

**Table 3:** Lesion conspicuity per reader, model, and input dose

Dose	Reader 1		Reader 2		*p*-value
	GAN	DDPM	GAN	DDPM	
5%	2 (2)	1 (1)	3 (2)	3 (2)	**0.007**
10%	3 (2)	3 (1)	4 (1)	5 (1)	1
25%	4 (2)	4 (2)	5 (1)	5 (0)	1

Values are stated as median (interquartile range). *p*-values represent selected pair-wise comparisons that were obtained from mixed-effects ordinal regression and subsequent estimation of estimated marginal means. During pair-wise comparison, Bonferroni correction was applied. Significant *p*-values are indicated in bold type. *DDPM* Denoising diffusion probabilistic model, *GAN* Generative adversarial network

### Comparable false positive lesion rate between both models, except at 5% dose

**Fig. 6:**
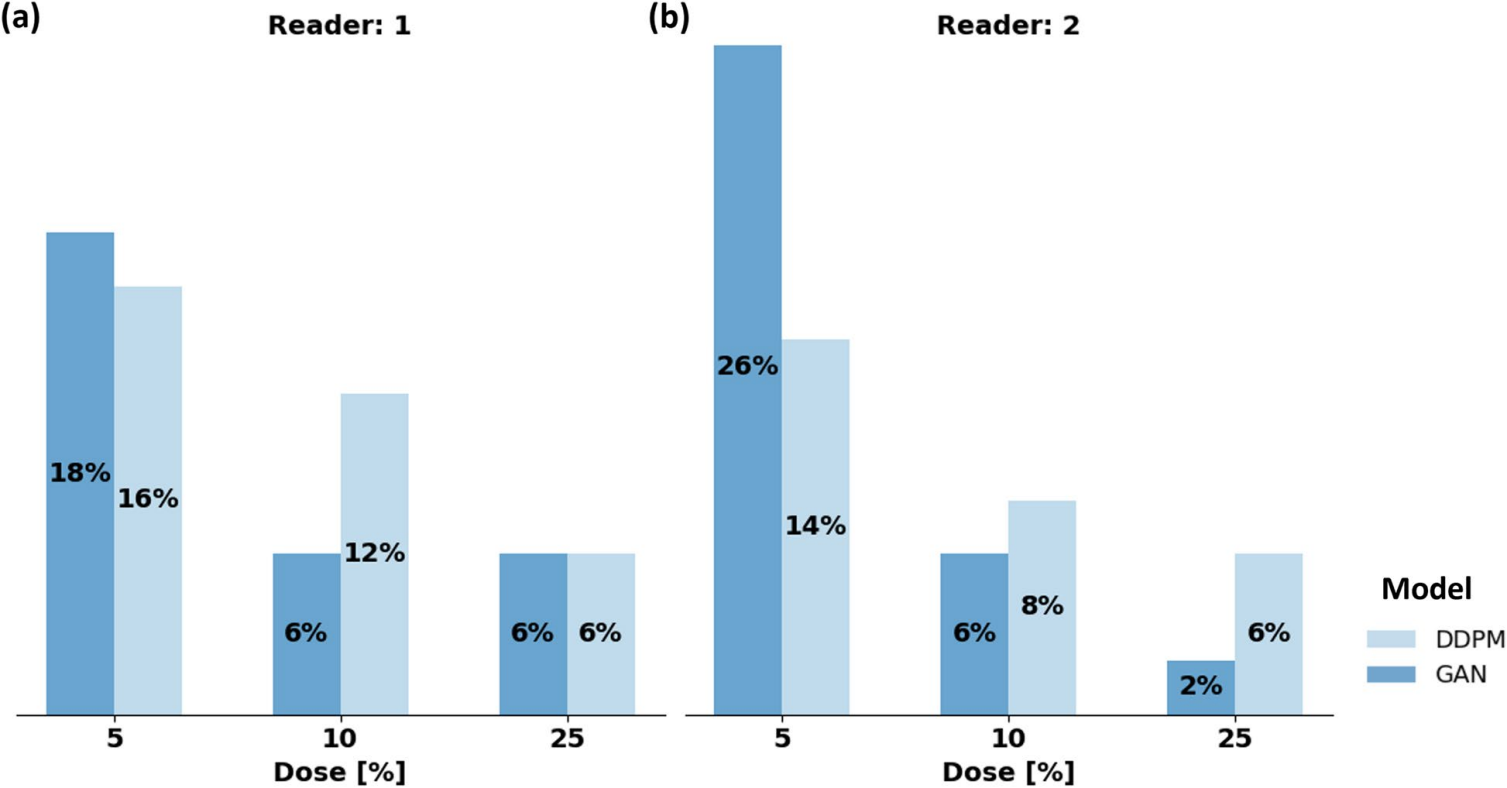
False positive lesion rates of the GAN and DDPM Model for reader 1 (left) and 2 (right). False positive rates refer to test set 2 that contained 50 lesion-free examinations at 5%, 10%, and 25% dose. Both readers were asked to state if a GAN- or DDPM-reconstructed MIP image contained at least one artificial lesion that could not be seen in the original MIP. *DDPM* Denoising diffusion probabilistic model, *GAN* Generative adversarial network, *MIP* Maximum intensity projection

**Fig. 7:**
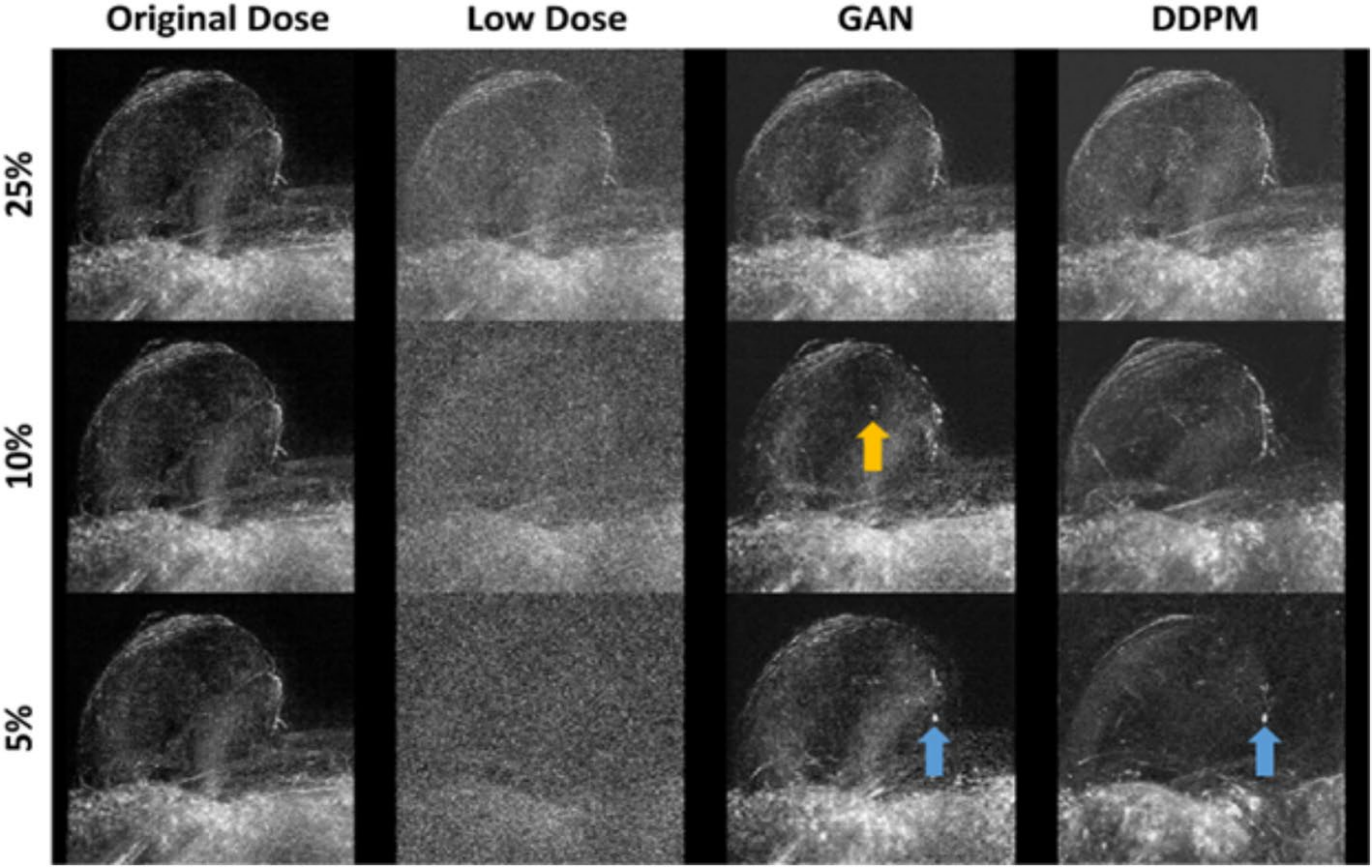
Image example from test set 2, showing an artificial (false-positive) lesion. MIP images of the right breast are presented. Both readers found false positive enhancements in the GAN and DDPM reconstructed images (blue arrow) at 5% dose. The faint enhancement in GAN 10% was not classified as a false positive finding by both readers (yellow arrow). Both readers found no false positive lesion at 10% and 25% dose in GAN and DDPM images. The false positive finding in GAN and DDPM at 5% could be misinterpreted as a small invasive breast cancer. *DDPM* Denoising diffusion probabilistic model, *GAN* Generative adversarial network

In the 50 lesion-free examinations, both readers observed false positive enhancements on the MIP images for all models and doses (Fig. 6). Following logistic regression, the type of model did not significantly influence the false positive rate (*p* = 1.000), but the dose did (*p* = 0.020). Supplementary Table S4 indicates complete statistical results. Accordingly, both readers observed an increased number of false positive enhancements, when the dose decreased from 25 to 5%. Figure 7 shows a lesion-free examination in which both readers observed a false-positive enhancement in the GAN and the DDPM reconstructed images at 5% dose. In the GAN and DDPM reconstructed images at 10% and 25% doses, no false-positive findings were found.

## Discussion

In this study, we trained a DDPM on virtual low-dose DCE subtraction images to recover the image contrast in breast MRI examinations for which only 25%, 10%, or 5% of the body-weight dependent GBCA dose would have been administered and compared it with GAN models. Visually, the reconstructed image quality decreased with decreasing GBCA dose for both model types, reflected by corresponding changes in quantitative image comparison metrics and lower lesion conspicuity scores. Two radiologists independently preferred the DDPM-reconstructed images at 25% dose while they preferred the GAN-reconstructed images at 5% dose. Lesion conspicuity between models was rated similar at 25% and 10% GBCA dose but significantly higher for the GAN at 5% dose. Here, the quantitative metrics also indicated better quality of the GAN-reconstructed images. At present, we cannot state superiority of one model type over the other throughout all investigated dose levels. While we trained a separate GAN model per investigated GBCA dose, the DDPM architecture can work with arbitrary GBCA doses.

We observed a higher lesion conspicuity in GAN-reconstructed images compared to DDPM-reconstructed images at 5% dose, coinciding with a preference of both readers for the former images. Dedicated training of the GAN models at 25%, 10%, and 5% doses may therefore allow for higher sensitivity with regard to lesions in the low-dose images. However, we also observed a higher false positive rate with the GAN compared to the DDPM when the dose was reduced to 5%, *i.e.*, the sensitivity increased while the specificity of the GAN decreased as compared to the DDPM.

Our results indicate that a contrast reduction below 25% is currently not possible using our models without a substantial increase in false-positive lesions and/or worsening of lesion conspicuity. We are not aware of any other study that has investigated the possibility to recover the image contrast based on different low doses. In contrast to the findings of this and our previous study [8], two studies in literature, *i.e.*, by Chung et al. [25] and by Wang et al. [13], reported the retrieval of contrast-enhanced MRI images of the breast based on precontrast images only. Of note, the four studies differ in several important aspects, *e.g.*, the type of DL model, the field strength, the number of cases employed for training (and, potentially, the different lesion types contained therein), the complexity of the breast protocol, and the kind of lesions contained in the test datasets. Both Chung et al. [24] and Wang et al. [13] used a full multiparametric breast MRI protocol including diffusion-weighted images, which could have led to a better capability to predict enhancement from precontrast images. Interestingly, both our previous study [8] and the study of Chung et al. [24] report consistently on a few failed, *i.e.*, missed, non-mass enhancements, when using only precontrast images for training. Wang et al. [13] do not report any missed lesions.

When reading MIP images, both radiologists noticed some false-positive findings for all models and dose levels. False positive findings on DL-reconstructed images are problematic because they require subsequent assessments (*e.g.*, rescheduling of the patient for a full-dose exam), which increases time spent until final diagnosis and raises patient concerns. Future research should therefore improve model performance with special emphasis on this aspect. For example, the addition of unenhanced images to the training process should be investigated, as they might prevent the creation of artificial lesions due to the added information content. The fact that no artificial lesions were reported for our previous study [8] (based on a GAN model trained at 25%, using both virtual low-dose subtraction images and native T1- and T2-weighted images) supports this assumption; however, care should be taken because that former study did not examine artificial findings in the same lesion-free test set utilized at present.

Quantitative measures are frequently employed in studies on artificial intelligence-based image generation to objectify comparisons between images; yet, they may diverge from radiologists’ impressions [26]. This was also the case for a GBCA dose of 25%, where the clear preference of the radiologists for DDPM-generated images was not reflected by any of the quantitative metrics. Potentially, the stronger noisy-looking background, visible for the DDPM-generated but not for most GAN-reconstructed and original images, led to worse performance of the quantitative metrics.

Both models have important methodological differences. While only one DDPM model in conjunction with a ResNet was needed to continuously cover the whole spectrum of GBCA dose levels (0 − 100%), we trained separate GANs per GBCA dose level (5%, 10%, and 25%). Therefore, a single DDPM could reconstruct original-dose images from various GBCA doses. Although an institution would likely adhere to a fixed, reduced dose level in practice, the same DDPM could still be used for data from different institutions that have chosen different GBCA doses.

It should be noted that risks associated with contrast agents such as nephrogenic systemic fibrosis or allergic reactions are generally low [27]. As far as macrocyclic GBCA are concerned, there is to date no evidence for their deposition in the brain, and even for linear GBCA, cerebral GBCA deposition has not been associated with any clinical side effects so far [28]. However, we believe that techniques to reduce the dose are highly relevant in breast MRI, especially when thinking towards its broader application in a screening setting. A reduced GBCA dose may increase acceptance of breast MRI among patients who are concerned about contrast agent administration. Furthermore, lower GBCA concentrations may enhance sensitivity to contrast agent dynamics, as demonstrated by Pineda et al. [7]. Additionally, a dose reduction would be relevant for economic and environmental reasons [29].

New contrast agents with higher relaxivity are another important approach to achieve these goals. For lesion assessment in contrast-enhanced body MRI, a dose of 0.05 mmol gadopiclenol per kg of body weight was shown to be non-inferior to a dose of 0.1 mmol gadobutrol per kg body weight [30]. It is important to note that high-relaxivity contrast agents can, in principle, be combined with DDPM- or GAN-based methods, thereby minimizing the administered dose.

Our study has limitations. First, our models were trained and tested on virtual low-dose subtraction images only. Although this allowed the models to be trained on several thousand examinations, the trained models would still need to be tested on real low-dose examinations. Unfortunately, such images were not available in our institution due to ethical concerns. Second, the test sets were of limited size, comprising only 50 single-sided breast examinations each. This choice was made to keep image readings for three different dose levels and two models within reasonable time limits. Third, we did not investigate any potentially beneficial effects of including the precontrast images into the models training, which could provide a useful route to decrease unwanted false-positive findings.

In conclusion, both GAN and DDPM have shown promising results in low-dose image reconstruction. Yet, neither model type yielded superior results over the other one for all tested dose levels and evaluation metrics. Further studies are needed to determine which of the two methods is better suited for GBCA reduction.

### Supplementary Information

**Additional file 1: Supplementary Table S1.** Scan Parameters. **Supplementary Table S2.** Analysis of numerical metrics by mixed effects linear regression, statistical results. **a–d** show the output of the lmer regression for the SSIM, PSNR, MSE and LPIPS metric, respectively. **e–h** show the respective pair-wise comparison results. *p*-value adjustment was performed using the Bonferroni method for 15 tests. *p*-values are stratified as ‘***’: *p* ≤ 0.001, ‘**’: *p* ≤ 0.01, ‘*’: *p* ≤ 0.05, ‘.’: *p* ≤ 0.1, ‘’: *p* > 0.1. Significant *p*-values are marked in bold text. Abbreviations: CI – confidence interval, L – linear term, Q – quadratic term. **Supplementary Table S3.** Analysis of Likert scores by mixed effects ordinal logistic regression, statistical results. **a** shows the output of the clmm regression. **b** shows the pair-wise comparison results. Results are average over the level of reader. *p*-value adjustment was performed with the Bonferroni method for 15 tests. *p*-values are stratified as ‘***’: *p* ≤ 0.001, ‘**’: *p* ≤ 0.01, ‘*’: *p* ≤ 0.05, ‘.’: *p* ≤ 0.1, ‘’: *p* > 0.1. Significant *p*-values are marked in bold text. Abbreviations: CI – confidence interval, L – linear term, Q – quadratic term. **Supplementary Table S4.** Analysis of false positive findings by mixed effects logistic regression, statistical results. **a** shows the output of the clmm regression. **b** shows the pair-wise comparison results. Results are average over the level of reader. *p*-value adjustment was performed with the Bonferroni method for 15 tests. *P*-values are stratified as ‘***’: *p* ≤ 0.001, ‘**’: *p* ≤ 0.01, ‘*’: *p* ≤ 0.05, ‘.’: *p* ≤ 0.1, ‘’: *p* > 0.1. Significant *p*-values are marked in bold text. Abbreviations: CI – confidence interval, L – linear term, Q – quadratic term.

## Data Availability

The datasets analyzed during the current study are available from the corresponding author on reasonable request.

## Abbreviations

CNR: Contrast-to-noise ratio
DCE: Dynamic contrast-enhanced
DDPM: Denoising diffusion probabilistic model
GAN: Generative adversarial network
GBCA: Gadolinium-based contrast agent
LPIPS: Learned perceptual image patch similarity
MIP: Maximum intensity projection
MSE: Mean squared error
PSNR: Peak signal-to-noise ratio
SSIM: Structural similarity index measure

## References

[1] Mann RM, Athanasiou A, Baltzer PAT (2022). Breast cancer screening in women with extremely dense breasts recommendations of the European Society of Breast Imaging (EUSOBI). Eur Radiol.

[2] Morris EA (2007). Diagnostic breast MR imaging: current status and future directions. Radiol Clin North Am.

[3] Kuhl CK, Schrading S, Strobel K (2014). Abbreviated breast magnetic resonance imaging (MRI): first postcontrast subtracted images and maximum-intensity projection—a novel approach to breast cancer screening With MRI. J Clin Oncol.

[4] Ramalho J, Castillo M, AlObaidy M (2015). High signal intensity in globus pallidus and dentate nucleus on unenhanced T1-weighted MR images: evaluation of two linear gadolinium-based contrast agents. Radiology.

[5] Akai H, Miyagawa K, Takahashi K (2021). Effects of gadolinium deposition in the brain on motor or behavioral function: a mouse model. Radiology.

[6] Melsaether AN, Kim E, Mema E (2019). Preliminary study: breast cancers can be well seen on 3T breast MRI with a half-dose of gadobutrol. Clin Imaging.

[7] Pineda F, Sheth D, Abe H (2019). Low-dose imaging technique (LITE) MRI: initial experience in breast imaging. Br J Radiol.

[8] Müller-Franzes G, Huck L, Tayebi Arasteh S (2023). Using machine learning to reduce the need for contrast agents in breast MRI through synthetic images. Radiology.

[9] Haase R, Pinetz T, Bendella Z (2023). Reduction of gadolinium-based contrast agents in MRI using convolutional neural networks and different input protocols: limited interchangeability of synthesized sequences with original full-dose images despite excellent quantitative performance. Invest Radiol.

[10] Gong E, Pauly JM, Wintermark M, Zaharchuk G (2018). Deep learning enables reduced gadolinium dose for contrast-enhanced brain MRI: deep learning reduces gadolinium dose. J Magn Reson Imaging.

[11] Luo H, Zhang T, Gong N-J (2021). Deep learning–based methods may minimize GBCA dosage in brain MRI. Eur Radiol.

[12] Pasumarthi S, Tamir JI, Christensen S (2021). A generic deep learning model for reduced gadolinium dose in contrast-enhanced brain MRI. Magn Reson Med.

[13] Wang P, Nie P, Dang Y (2021). Synthesizing the first phase of dynamic sequences of breast MRI for Enhanced lesion identification. Front Oncol.

[14] Ho  J, Jain  A, Abbeel  P (2020). Denoising diffusion probabilistic models.

[15] Kuhl CK, Strobel K, Bieling H (2017). Supplemental breast MR imaging screening of women with average risk of breast cancer. Radiology.

[16] Nichol A, Dhariwal P (2021). Improved denoising diffusion probabilistic models.

[17] Loshchilov  I, Hutter F (2017). Decoupled weight decay regularization.

[18] He K, Zhang X, Ren S, Sun J (2016). Deep residual learning for image recognition. 2016 IEEE Conference on Computer Vision and Pattern Recognition (CVPR).

[19] Isola P, Zhu J-Y, Zhou T, Efros AA (2017). Image-to-image translation with conditional adversarial networks. 2017 IEEE Conference on Computer Vision and Pattern Recognition (CVPR).

[20] Kingma DP, Ba  J (2017). Adam: a method for stochastic optimization.

[21] Wang T-C, Liu M-Y, Zhu J-Y (2018). High-resolution image synthesis and semantic manipulation with conditional GANs. 2018 IEEE/CVF Conference on Computer Vision and Pattern Recognition.

[22] Wang Z, Bovik AC, Sheikh HR, Simoncelli EP (2004). Image quality assessment: from error visibility to structural similarity. IEEE Trans Image Process.

[23] Zhang R, Isola P, Efros AA (2018). The unreasonable effectiveness of deep features as a perceptual metric.

[24] R: Bonferroni correction. https://search.r-project.org/CRAN/refmans/mutoss/html/bonferroni.html. Accessed 13 Feb 2024

[25] Chung M, Calabrese E, Mongan J (2023). Deep learning to simulate contrast-enhanced breast MRI of invasive breast cancer. Radiology.

[26] Mason A, Rioux J, Clarke SE (2020). Comparison of objective image quality metrics to expert radiologists’ scoring of diagnostic quality of MR images. IEEE Trans Med Imaging.

[27] Costelloe CM, Amini B, Madewell JE (2020). Risks and benefits of gadolinium-based contrast-enhanced MRI. Semin Ultrasound CT MRI.

[28] Van Der Molen AJ, Quattrocchi CC, Mallio CA (2023). Ten years of gadolinium retention and deposition: ESMRMB-GREC looks backward and forward. Eur Radiol.

[29] Brünjes R, Hofmann T (2020). Anthropogenic gadolinium in freshwater and drinking water systems. Water Res.

[30] Kuhl C, Csőszi T, Piskorski W (2023). Efficacy and safety of half-dose gadopiclenol versus full-dose gadobutrol for contrast-enhanced body MRI. Radiology.

