# Supplementary material for: Diffusion probabilistic versus generative adversarial models to reduce contrast agent dose in breast MRI
Source: Eur Radiol Exp. 2024 May 1;8:53. doi: 10.1186/s41747-024-00451-3 (PMC11061055; doi:10.1186/s41747-024-00451-3)
Supplement: Supplementary file 1 — Additional file 1: Supplementary Table S1. Scan Parameters. Supplementary Table S2. Analysis of numerical metrics by mixed effects linear regression, statistical results. a–d show the output of the lmer regression for the SSIM, PSNR, MSE and LPIPS metric, respectively. e–h show the respective pair-wise comparison results. p-value adjustment was performed using the Bonferroni method for 15 tests. p-values are stratified as ‘***’: p ≤ 0.001, ‘**’: p ≤ 0.01, ‘*’: p ≤ 0.05, ‘.’: p ≤ 0.1, ‘’: p > 0.1. Significant p-values are marked in bold text. Abbreviations: CI – confidence interval, L – linear term, Q – quadratic term. Supplementary Table S3. Analysis of Likert scores by mixed effects ordinal logistic regression, statistical results. a shows the output of the clmm regression. b shows the pair-wise comparison results. Results are average over the level of reader. p-value adjustment was performed with the Bonferroni method for 15 tests. p-values are stratified as ‘***’: p ≤ 0.001, ‘**’: p ≤ 0.01, ‘*’: p ≤ 0.05, ‘.’: p ≤ 0.1, ‘’: p > 0.1. Significant p-values are marked in bold text. Abbreviations: CI – confidence interval, L – linear term, Q – quadratic term. Supplementary Table S4. Analysis of false positive findings by mixed effects logistic regression, statistical results. a shows the output of the clmm regression. b shows the pair-wise comparison results. Results are average over the level of reader. p-value adjustment was performed with the Bonferroni method for 15 tests. P-values are stratified as ‘***’: p ≤ 0.001, ‘**’: p ≤ 0.01, ‘*’: p ≤ 0.05, ‘.’: p ≤ 0.1, ‘’: p > 0.1. Significant p-values are marked in bold text. Abbreviations: CI – confidence interval, L – linear term, Q – quadratic term. [file 41747_2024_451_MOESM1_ESM.pdf]

Diffusion Probabilistic Models versus Generative Adversarial Models to Reduce Contrast Agent Dose in Breast MRI

ELECTRONIC SUPPLEMENTARY MATERIAL

Supplementary Table S1: Scan Parameters

Scanner	Philips (Achieva and Ingenia)	
Orientation	Axial	
Field Strength [T]	1.5	
Sequence	T1w dynamic	T2w
Acquisition Type	2D	2D
Echo Type	Gradient Echo	Turbo Spin Echo
Fat Suppression	No	No
TR [ms]	264 ± 22	4008 ± 201
TE [ms]	4.6 ± 0.1	110
Flip angle [°]	90 ± 3	90
Slice Thickness [mm]	3.1 ± 0.2	3.1 ± 0.2
Number of slices	28 ± 2	33 ± 2
Matrix X	520 ± 22	535 ± 39
Matrix Y	520 ± 22	535 ± 39
Field of View X [mm]	333 ± 26	332 ± 26
Field of View Y [mm]	333 ± 26	332 ± 26

Supplementary Table S2

Supplementary Table S2: Analysis of numerical metrics by mixed effects linear regression, statistical results. (a) – (d) show the output of the lmer regression for the SSIM, PSNR, MSE and LPIPS metric, respectively. (e) – (h) show the respective pair-wise comparison results. P-value adjustment was performed using the Bonferroni method for 15 tests. P-values are stratified as '***': P≤0.001, '**': P≤0.01, '*': P≤0.05, '.': P≤0.1, ' ': P>0.1. Significant p-values are marked in **bold text**. Abbreviations: CI – confidence interval, L – linear term, Q – quadratic term.

(a) SSIM – model output

**Fixed effects**	**Estimate**	**Std. Error**	**df**	**z value**	**Pr(>|z|)**	**P**	**CI 2.50%**	**CI 97.50%**
(Intercept)	0.50405	0.01495	50.3	33.707	**< 2e-16**	***	0.4745	0.5336
dose.L	0.12794	0.00417	245	30.662	**< 2e-16**	***	0.1198	0.1361
dose.Q	0.03548	0.00417	245	8.504	**1.83E-15**	***	0.0274	0.0436
modelGAN	-0.00646	0.00341	245	-1.895	0.0593	.	-0.0131	0.0002
dose.L:modelGAN	0.01464	0.0059	245	2.481	**0.0138**	*	0.0031	0.0261
dose.Q:modelGAN	-0.01437	0.0059	245	-2.435	**0.0156**	*	-0.0259	-0.0029

(b) PSNR – model output

**Fixed effects**	**Estimate**	**Std. Error**	**df**	**z value**	**Pr(>|z|)**	**P**	**CI 2.50%**	**CI 97.50%**
(Intercept)	21.6734	0.3952	76.9	54.847	**< 2e-16**	***	20.897	22.450
dose.L	3.005	0.4404	245	6.823	**6.94E-11**	***	2.1472	3.8628
dose.Q	1.1824	0.4404	245	2.685	**0.00775**	**	0.3246	2.0403
modelGAN	2.5062	0.3596	245	6.969	**2.93E-11**	***	1.8058	3.2067
dose.L:modelGAN	-0.1857	0.6229	245	-0.298	0.76586		-1.3988	1.0275
dose.Q:modelGAN	-0.7778	0.6229	245	-1.249	0.21293		-1.991	0.4353

(c) MSE – model output

**Fixed effects**	**Estimate**	**Std. Error**	**df**	**z value**	**Pr(>|z|)**	**P**	**CI 2.50%**	**CI 97.50%**
(Intercept)	94.076	1.826	105.5	51.507	**< 2e-16**	***	90.500	97.653
dose.L	-6.237	2.59	245	-2.408	**0.0168**	*	-11.283	-1.1921
dose.Q	-4.016	2.59	245	-1.55	0.1223		-9.0615	1.0291
modelGAN	-14.637	2.115	245	-6.921	**3.91E-11**	***	-18.757	-10.518
dose.L:modelGAN	-4.659	3.663	245	-1.272	0.2046		-11.794	2.4757
dose.Q:modelGAN	2.96	3.663	245	0.808	0.4198		-4.175	10.095

(d) LPIPS – model output

**Fixed effects**	**Estimate**	**Std. Error**	**df**	**z value**	**Pr(>|z|)**	**P**	**CI 2.50%**	**CI 97.50%**
(Intercept)	0.36472	0.01576	73.5	23.136	**< 2e-16**	***	0.3337	0.3957
dose.L	-0.10061	0.01674	245	-6.009	**6.71E-09**	***	-0.1332	-0.068
dose.Q	-0.03105	0.01674	245	-1.855	0.0648	.	-0.0637	0.0016
modelGAN	-0.11058	0.01367	245	-8.089	**2.80E-14**	***	-0.1372	-0.0839
dose.L:modelGAN	0.05788	0.02368	245	2.445	**0.0152**	*	0.0118	0.104
dose.Q:modelGAN	0.02983	0.02368	245	1.26	0.209		-0.0163	0.0759

(e) SSIM – pair-wise comparisons

contrast	estimate	SE	df	z.ratio	p.value
dose5 DDPM - dose10 DDPM	-0.04701	0.0059	245	-7.966	**<.0001**
dose5 DDPM - dose25 DDPM	-0.18093	0.0059	245	-30.662	**<.0001**
dose5 DDPM - dose5 GAN	0.02267	0.0059	245	3.842	**0.0023**
dose5 DDPM - dose10 GAN	-0.05228	0.0059	245	-8.861	**<.0001**
dose5 DDPM - dose25 GAN	-0.17896	0.0059	245	-30.328	**<.0001**
dose10 DDPM - dose25 DDPM	-0.13392	0.0059	245	-22.695	**<.0001**
dose10 DDPM - dose5 GAN	0.06968	0.0059	245	11.808	**<.0001**
dose10 DDPM - dose10 GAN	-0.00528	0.0059	245	-0.894	1
dose10 DDPM - dose25 GAN	-0.13195	0.0059	245	-22.361	**<.0001**
dose25 DDPM - dose5 GAN	0.2036	0.0059	245	34.504	**<.0001**
dose25 DDPM - dose10 GAN	0.12864	0.0059	245	21.801	**<.0001**
dose25 DDPM - dose25 GAN	0.00197	0.0059	245	0.334	1
dose5 GAN - dose10 GAN	-0.07496	0.0059	245	-12.703	**<.0001**
dose5 GAN - dose25 GAN	-0.20163	0.0059	245	-34.17	**<.0001**
dose10 GAN - dose25 GAN	-0.12667	0.0059	245	-21.467	**<.0001**

(f) PSNR – pair-wise comparisons

contrast	estimate	SE	df	z.ratio	p.value
dose5 DDPM - dose10 DDPM	-0.677	0.623	245	-1.086	1
dose5 DDPM - dose25 DDPM	-4.25	0.623	245	-6.823	**<.0001**
dose5 DDPM - dose5 GAN	-2.32	0.623	245	-3.725	**0.0036**
dose5 DDPM - dose10 GAN	-3.818	0.623	245	-6.13	**<.0001**
dose5 DDPM - dose25 GAN	-6.307	0.623	245	-10.126	**<.0001**
dose10 DDPM - dose25 DDPM	-3.573	0.623	245	-5.737	**<.0001**
dose10 DDPM - dose5 GAN	-1.643	0.623	245	-2.638	0.133
dose10 DDPM - dose10 GAN	-3.141	0.623	245	-5.043	**<.0001**
dose10 DDPM - dose25 GAN	-5.63	0.623	245	-9.04	**<.0001**
dose25 DDPM - dose5 GAN	1.93	0.623	245	3.098	**0.0326**
dose25 DDPM - dose10 GAN	0.432	0.623	245	0.693	1
dose25 DDPM - dose25 GAN	-2.057	0.623	245	-3.303	**0.0165**
dose5 GAN - dose10 GAN	-1.498	0.623	245	-2.405	0.2537
dose5 GAN - dose25 GAN	-3.987	0.623	245	-6.401	**<.0001**
dose10 GAN - dose25 GAN	-2.489	0.623	245	-3.996	**0.0013**

(g) MSE – pair-wise comparisons

contrast	estimate	SE	df	z.ratio	p.value
dose5 DDPM - dose10 DDPM	-0.508	3.66	245	-0.139	1
dose5 DDPM - dose25 DDPM	8.821	3.66	245	2.408	0.2517
dose5 DDPM - dose5 GAN	10.134	3.66	245	2.766	0.0915
dose5 DDPM - dose10 GAN	16.546	3.66	245	4.517	**0.0001**
dose5 DDPM - dose25 GAN	25.545	3.66	245	6.973	**<.0001**

dose10 DDPM - dose25 DDPM	9.329	3.66	245	2.547	0.1723
dose10 DDPM - dose5 GAN	10.643	3.66	245	2.905	**0.0601**
dose10 DDPM - dose10 GAN	17.054	3.66	245	4.656	**0.0001**
dose10 DDPM - dose25 GAN	26.053	3.66	245	7.112	**<.0001**
dose25 DDPM - dose5 GAN	1.313	3.66	245	0.359	1
dose25 DDPM - dose10 GAN	7.725	3.66	245	2.109	0.5397
dose25 DDPM - dose25 GAN	16.724	3.66	245	4.565	**0.0001**
dose5 GAN - dose10 GAN	6.412	3.66	245	1.75	1
dose5 GAN - dose25 GAN	15.41	3.66	245	4.207	**0.0005**
dose10 GAN - dose25 GAN	8.999	3.66	245	2.456	0.2209

(h) LPIPS – pair-wise comparisons

**contrast**	**estimate**	**SE**	**df**	**z.ratio**	**p.value**
dose5 DDPM - dose10 DDPM	0.03311	0.0237	245	1.398	1
dose5 DDPM - dose25 DDPM	0.14228	0.0237	245	6.009	**<.0001**
dose5 DDPM - dose5 GAN	0.13933	0.0237	245	5.884	**<.0001**
dose5 DDPM - dose10 GAN	0.16804	0.0237	245	7.097	**<.0001**
dose5 DDPM - dose25 GAN	0.19975	0.0237	245	8.436	**<.0001**
dose10 DDPM - dose25 DDPM	0.10917	0.0237	245	4.611	**0.0001**
dose10 DDPM - dose5 GAN	0.10622	0.0237	245	4.486	**0.0002**
dose10 DDPM - dose10 GAN	0.13493	0.0237	245	5.699	**<.0001**
dose10 DDPM - dose25 GAN	0.16664	0.0237	245	7.038	**<.0001**
dose25 DDPM - dose5 GAN	-0.00296	0.0237	245	-0.125	1
dose25 DDPM - dose10 GAN	0.02576	0.0237	245	1.088	1
dose25 DDPM - dose25 GAN	0.05747	0.0237	245	2.427	**0.2391**
dose5 GAN - dose10 GAN	0.02871	0.0237	245	1.213	1
dose5 GAN - dose25 GAN	0.06043	0.0237	245	2.552	**0.1697**
dose10 GAN - dose25 GAN	0.03171	0.0237	245	1.339	1

Supplementary Table S3

Supplementary Table S3: Analysis of Likert scores by mixed effects ordinal logistic regression, statistical results. (a) shows the output of the clmm regression. (b) shows the pair-wise comparison results. Results are average over the level of reader. P-value adjustment was performed with the Bonferroni method for 15 tests. P-values are stratified as '***': P≤0.001, '**': P≤0.01, '*': P≤0.05, '.': P≤0.1, ' ': P>0.1. Significant p-values are marked in **bold text**. Abbreviations: CI – confidence interval, L – linear term, Q – quadratic term.

(a) model output

Coefficients	Estimate	Std. Error	z value	Pr(>|z|)	P	CI 2.50%	CI 97.50%
dose.L	3.487	0.2572	13.558	**< 2e-16**	***	2.9830	3.9911
dose.Q	-0.1619	0.2004	-0.808	0.41914		-0.5546	0.2308
modelGAN	0.2553	0.1668	1.53	0.12597		-0.0717	0.5823
reader2	2.6067	0.1977	13.186	**< 2e-16**	***	2.2192	2.994
dose.L:modelGAN	-0.8945	0.2983	-2.999	**0.00271**	**	-1.4791	-0.3100
dose.Q:modelGAN	0.2274	0.2799	0.812	0.41659		-0.3212	0.7759

(b) pair-wise comparisons

contrast	estimate	SE	df	z.ratio	p.value
dose5 DDPM - dose10 DDPM	-2.664	0.296	Inf	-8.995	**<.0001**
dose5 DDPM - dose25 DDPM	-4.9314	0.364	Inf	-13.558	**<.0001**
dose5 DDPM - dose5 GAN	-0.9806	0.281	Inf	-3.493	**0.0072**
dose5 DDPM - dose10 GAN	-2.7336	0.297	Inf	-9.192	**<.0001**
dose5 DDPM - dose25 GAN	-4.647	0.349	Inf	-13.309	**<.0001**
dose10 DDPM - dose25 DDPM	-2.2674	0.314	Inf	-7.21	**<.0001**
dose10 DDPM - dose5 GAN	1.6833	0.283	Inf	5.957	**<.0001**
dose10 DDPM - dose10 GAN	-0.0697	0.272	Inf	-0.256	1
dose10 DDPM - dose25 GAN	-1.983	0.302	Inf	-6.563	**<.0001**
dose25 DDPM - dose5 GAN	3.9507	0.342	Inf	11.548	**<.0001**
dose25 DDPM - dose10 GAN	2.1978	0.312	Inf	7.05	**<.0001**
dose25 DDPM - dose25 GAN	0.2844	0.313	Inf	0.908	1
dose5 GAN - dose10 GAN	-1.753	0.283	Inf	-6.198	**<.0001**
dose5 GAN - dose25 GAN	-3.6663	0.328	Inf	-11.177	**<.0001**
dose10 GAN - dose25 GAN	-1.9133	0.299	Inf	-6.396	**<.0001**

Supplementary Table S4

Supplementary Table S4: Analysis of false positive findings by mixed effects logistic regression, statistical results. (a) shows the output of the clmm regression. (b) shows the pair-wise comparison results. Results are average over the level of reader. P-value adjustment was performed with the Bonferroni method for 15 tests. P-values are stratified as '***': P≤0.001, '**': P≤0.01, '*': P≤0.05, '.': P≤0.1, ' ': P>0.1. Significant p-values are marked in **bold text**. Abbreviations: CI – confidence interval, L – linear term, Q – quadratic term.

(a) model output

**Coefficients**	**Estimate**	**Std. Error**	**z value**	**Pr(>|z|)**	**P**	**CI 2.50%**	**CI 97.50%**
dose.L	-0.97344	0.42211	-2.306	**0.0211**	*	2.2848	4.0865
dose.Q	-0.05038	0.39766	-0.127	0.8992		-1.8008	-0.1461
modelGAN	-0.23002	0.36439	-0.631	0.5279		-0.8298	0.729
reader2	-0.05099	0.31943	-0.16	0.8732		-0.9442	0.4842
dose.L:modelGAN	-0.87593	0.63403	-1.382	0.1671		-0.6771	0.5751
dose.Q:modelGAN	0.6369	0.62854	1.013	0.3109		-2.1186	0.3667

(b) pair-wise comparisons

**contrast**	**estimate**	**SE**	**df**	**z.ratio**	**p.value**
dose5 DDPM - dose10 DDPM	0.6270	0.508	Inf	1.233	1
dose5 DDPM - dose25 DDPM	1.3800	0.597	Inf	2.306	0.3165
dose5 DDPM - dose5 GAN	-0.6490	0.437	Inf	-1.487	1
dose5 DDPM - dose10 GAN	1.3800	0.597	Inf	2.306	0.3165
dose5 DDPM - dose25 GAN	1.9700	0.701	Inf	2.804	0.0758
dose10 DDPM - dose25 DDPM	0.7500	0.628	Inf	1.194	1
dose10 DDPM - dose5 GAN	-1.2800	0.487	Inf	-2.618	0.1326
dose10 DDPM - dose10 GAN	0.7500	0.628	Inf	1.194	1
dose10 DDPM - dose25 GAN	1.3400	0.726	Inf	1.846	0.9733
dose25 DDPM - dose5 GAN	-2.0300	0.582	Inf	-3.48	**0.0075**
dose25 DDPM - dose10 GAN	0.0000	0.696	Inf	0	1
dose25 DDPM - dose25 GAN	0.5890	0.781	Inf	0.754	1
dose5 GAN - dose10 GAN	2.0300	0.582	Inf	3.48	**0.0075**
dose5 GAN - dose25 GAN	2.6200	0.691	Inf	3.786	**0.0023**
dose10 GAN - dose25 GAN	0.5890	0.781	Inf	0.754	1
